# Anticancer Potentials of the Lignan Magnolin: A Systematic Review

**DOI:** 10.3390/molecules28093671

**Published:** 2023-04-23

**Authors:** Md. Shimul Bhuia, Polrat Wilairatana, Raihan Chowdhury, Asraful Islam Rakib, Hossam Kamli, Ahmad Shaikh, Henrique D. M. Coutinho, Muhammad Torequl Islam

**Affiliations:** 1 Department of Pharmacy, Bangabandhu Sheikh Mujibur Rahman Science and Technology University, Gopalganj 8100, Bangladesh; shimulbhuia.pharm@gmail.com (M.S.B.);; 2 Department of Clinical Tropical Medicine, Faculty of Tropical Medicine, Mahidol University, Bangkok 10400, Thailand; 3 Department of Clinical Laboratory Sciences, College of Applied Medical Sciences, King Khalid University, Abha 61421, Saudi Arabia; 4 Department of Biological Chemistry, Regional University of Cariri, Crato 63105-000, CE, Brazil

**Keywords:** lignan, magnolin, biological sources, pharmacokinetic profile, cancer cell lines

## Abstract

Magnolin is a naturally occurring, multi-bioactive lignan molecule with inherent anticancer effects. This study aims to summarize the botanical origins and anticancer properties of magnolin. For this, a recent (as of March 2023) literature review was conducted using various academic search engines, including PubMed, Springer Link, Wiley Online, Web of Science, Science Direct, and Google Scholar. All the currently available information about this phytochemical and its role in various cancer types has been gathered and investigated. Magnolin is a compound found in many different plants. It has been demonstrated to have anticancer activity in numerous experimental models by inhibiting the cell cycle (G1 and G2/M phase); inducing apoptosis; and causing antiinvasion, antimetastasis, and antiproliferative effects via the modulation of several pathways. In conclusion, magnolin showed robust anticancer activity against many cancer cell lines by altering several cancer signaling pathways in various non- and pre-clinical experimental models, making it a promising plant-derived chemotherapeutic option for further clinical research.

## 1. Introduction

Cancer is a broad collection of illnesses that can begin in practically any organ or tissue of the body [1]. The World Health Organization (WHO) stated that approximately 10 million cancer-related deaths occurred worldwide in 2020, accounting for nearly one-sixth of all deaths. Furthermore, around 400,000 children are diagnosed with cancer each year (https://www.who.int/health-topics/cancer, accessed on 5 March 2023). These diseases occur when atypical cells multiply uncontrollably, invade neighboring tissues beyond their usual boundaries, and metastasize to other organs [2]. The underlying anomaly that leads to the formation of cancer is the persistent and uncontrolled growth of cancerous cells [3]. Cancer cells do not behave in the same way as normal cells, and they do not respond appropriately to the signals that govern normal cell behavior. Instead, cancer cells grow and divide without regulation, invade healthy tissues and organs, and eventually metastasize to other parts of the body [4]. The process of cancer development is seen as a complex series of steps at the cellular level, which includes mutation and selection for cells with enhanced abilities for proliferation, survival, invasion, and metastasis. These steps occur progressively, with cells acquiring more and more of these traits over time [5]. The standard process of the biological system can be changed by daily exposure to dangerous toxicants, which eventually results in genetic damage [6]. The development of cancer is a multifaceted process that occurs over a series of steps, as we know, and as a result, numerous factors may influence the likelihood of cancer development. Therefore, it is overly simplistic to attribute most cancers to a single cause [7]. Despite the complexity of cancer development, several factors, such as radiation, viruses, and chemicals, have been identified to cause cancer in both humans and laboratory animals. These agents have been found to induce cancer through various mechanisms [8,9,10]. A poor diet with insufficient fruits and vegetables and lifestyle choices such as smoking and drinking alcohol can also raise the risk of developing cancer [11,12].

The treatment plan for cancer is determined based on several factors, such as the type and stage of the cancer, as well as the patient’s overall health and age. Treatment options may include surgery, radiation therapy, immunotherapy, chemotherapy, and targeted therapy. Early detection and treatment can elevate the chances of survival and reduce the risk of complications [13,14,15]. For the majority of human cancers, chemotherapy offers variable levels of effectiveness as a treatment while offering curative care for specific cancer types [16,17]. Current anticancer drugs have multiple limitations, including resistance, toxicity, limited efficacy, and cost. Cancer cells can develop resistance to anticancer drugs, which hinders their effectiveness. Additionally, many anticancer drugs cause significant side effects that impact patients’ quality of life. A lack of specificity can also cause healthy cells to be affected, leading to toxicity and side effects [18,19,20]. This is why we need to develop more safe and effective novel anticancer drugs. 

Natural remedies are becoming increasingly prevalent worldwide as conventional or supplementary treatments for curable and incurable diseases and for encouraging health and disease prevention [21]. Due to their negligible side effects and good safety records, they are widely used in various diseases as effective therapeutic routes [22]. Lead compounds are abundant in plants and are used in drug discovery. Most (60–70%) of the antibacterial and anticancer medications used in clinical settings are natural compounds or their derivatives [23,24].

Naturally occurring lignans are a group of secondary metabolites that are found in a variety of sources, such as seeds, nuts, cereals, vegetables, and fruits. Lignans serve a range of purposes in plants, and their multifaceted functions are significant for various organisms, including humans [25,26,27]. They are mainly liable for the defense mechanism and are distinguished by the presence of two terminal phenyl groups in their chemical structure [28]. Phytochemicals that belong to the lignan class exhibit diverse biological features, including antioxidant, anti-inflammatory, antiestrogenic, anticarcinogenic, and antitumor activities [29,30]. Several epidemiological studies have suggested that lignans may lower the risk of cardiovascular disease. However, the impact of lignans on other chronic ailments, such as breast cancer, is still debated [31]. Additionally, the dietary intake of lignans has been linked with a diminished risk of other types of cancer, including colon cancer, gastric adenocarcinoma, and esophageal carcinoma. However, there have been very few human studies conducted on this topic [32]. The scientific community has previously mentioned the discovery and development of lignan derivatives for the creation of anticancer therapeutics. Examples of such drugs include etoposide, teniposide, and etopophos [33].

**Figure 1:**
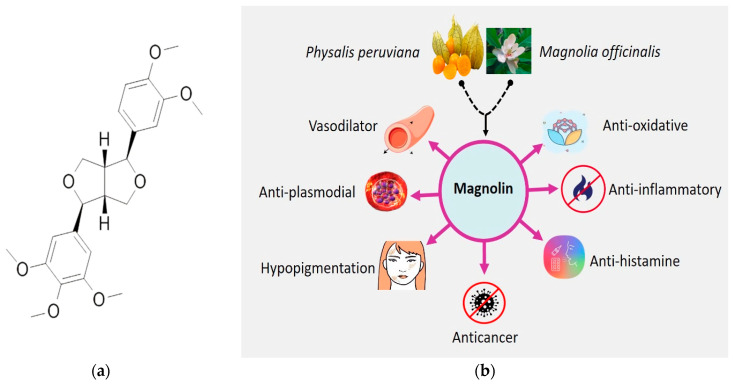
(**a**) Two-dimensional chemical structure of magnolin and (**b**) different pharmacological activities of magnolin based on literature.

**Figure 2:**
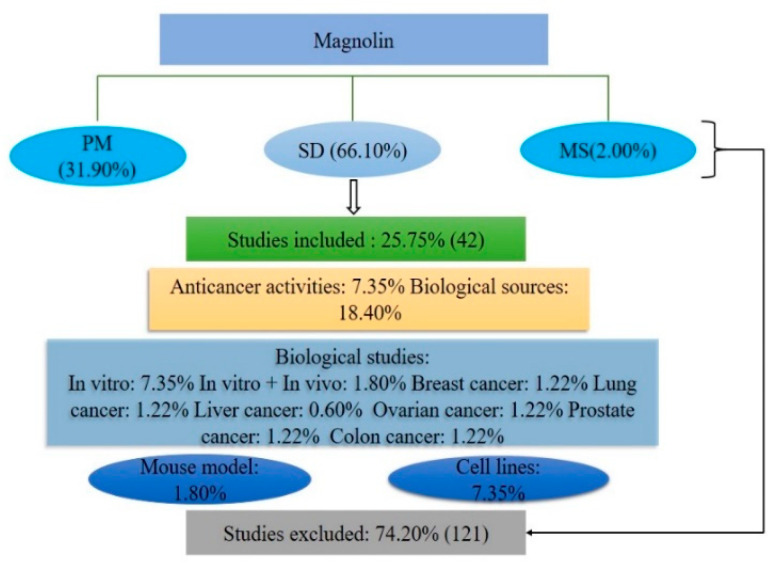
Schematic presentation of data collection, inclusion and exclusion criteria (PM: PubMed; SD: Science Direct; MS: miscellaneous (e.g., Google Scholar and Research Gate)).

Magnolin is a lignan (C_23_H_28_O_7_, Figure 1), chemically known as (3S,3aR,6S,6aR)-3-(3,4-dimethoxy phenyl)-6-(3,4,5-tri methoxyphenyl)-1,3,3a,4,6,6a-hexahydrofuro[3,4-c] furan, that has been isolated from the Magnolia genus [34,35], which is a large genus of about 210 flowering species in the family Magnoliaceae [36], and has been shown to have health-enhancing properties (Figure 2) in in vivo and in vitro test systems [37,38,39,40,41,42,43,44]. Magnolin exhibits various pharmacological activities, including anti-oxidative, anti-inflammatory, and vasodilatory effects. It has also been found to have protective effects against contrast-induced nephropathy and has been shown to inhibit the migration and invasion of lung cancer cells [41,43]. Additionally, magnolin has been found to be capable of suppressing cell proliferation and transformation by suppressing the ERKs/RSK2 signaling pathways and impairing the G1/S cell-cycle transition [45]. This review focuses on magnolin’s general anticancer mechanism. For this goal, we have compiled the most recent information on this optimistic anticancer agent’s anticancer activity.

## 2. Results and Discussion

### 2.1. Database Reports

A total of 163 pieces of evidence were identified through the databases of PubMed and Science Direct, as well as through other sources such as Google Scholar and Research Gate. These databases covered 31.90, 66.10, and 2.00% of the evidence, respectively. Based on the inclusion criteria, this study includes 42 reports. Among them, 7.35% of the reports pertain to the anticancer activities of magnolin, while 18.40% of the reports pertain to its botanical sources, pharmacological activities, and pharmacokinetic features. Among the different cancers, 1.22, 1.22, 1.22, 0.6, 1.22, 1.22, and 0.6% of the cases correspond to breast cancer, lung cancer, ovarian cancer, liver cancer, prostate cancer, colon cancer, and pancreatic carcinoma, respectively. Meanwhile, 1.80% of the reports correspond to mouse models, and 7.35% correspond to in vitro experiments (investigated on different cell lines). After reviewing the titles, abstracts, and contents of the reports, 121 were deemed irrelevant to the study and were subsequently excluded. A logical flowchart for this study is exhibited in Figure 2.

### 2.2. Botanical Sources

Medicinal plants are considered significant resources for discovering novel drugs worldwide [46,47]. They hold cultural and economic importance to local communities and have been utilized in traditional and popular medicine as well as in the development of pharmaceuticals [48,49]. The commercial and scientific interest in medicinal plants as a source of primary materials for the herbal pharmaceutical industries is increasing rapidly due to the growing global demand [50]. The World Health Organization (WHO) reports that a significant proportion of individuals in developing countries (70–95%) rely on medicinal plants for their primary healthcare needs. However, despite their widespread use, only a small fraction (15%) of medicinal plants worldwide has been analyzed to measure their potential therapeutic benefits based on their phytochemical and phytopharmacological properties [51]. Magnolin, a biologically active compound, has been extracted from plants of the Magnolia genus [35]. Three species of magnolia, including Magnolia denudata, Magnolia sprengeri, and Magnolia biondii, have been recognized in the pharmacopoeia and are known for their therapeutic benefits [42]. The compound source is a component of the flowers, leaves, seeds, roots, and aerial portions of numerous species, according to accounts in the literature. The botanical origins of this lignan are shown in Table 1.

### 2.3. Cellular and Molecular Anticancer Mechanisms of Magnolin

New precision medicine treatments for cancer are developed by utilizing information obtained from changes that occur at the molecular level in cancer genes and their associated signaling pathways. These days, it is widely acknowledged that signaling pathways and molecular networks play crucial roles in carrying out and regulating vital cellular processes that promote cell survival and growth and are hence largely responsible for the development of cancer as well as its potential treatment [77]. In cancer, two pathways, namely, the PI3K/AKT/mTOR signal transduction pathway and the Ras/MEK pathway, are commonly activated or mutated [78]. These pathways are closely interlinked in transmitting signals from receptor tyrosine kinases (RTKs) to intracellular effector proteins and cell cycle regulators, thus regulating upstream cellular signals [79]. Some other important signaling pathways that develop cancer and can be the target of therapeutic development include cell cycle, Hippo signaling [80], Notch signaling [81], Myc signaling [82,83], oxidative stress response/Nrf2 [84,85], TGFb signaling [86], β-catenin/Wnt signaling [87], and p53 signaling pathways [88]. CDK4/6 inhibitors (CDK4/6i) have made significant advancements in cancer treatment among all the signaling pathways targeting CDK4/6 activation [89].

Due to the growing number of deaths caused by cancer, it is essential to develop therapeutic strategies that have fewer cytotoxic side effects and are less prone to resistance. Natural compounds have been found to possess multiple beneficial activities, such as promoting overall health and providing cancer treatment [90]. Natural products have demonstrated preferential advantages against cancer cells when compared to normal cells. Additionally, their chemical structures can serve as models for the development of innovative drugs [90,91]. Using these models, drugs can be formulated that offer comparable or superior benefits to those provided by natural products. Moreover, these drugs may have fewer side effects and lower chances of resistance compared to the original natural products [92,93].

**Figure 3:**
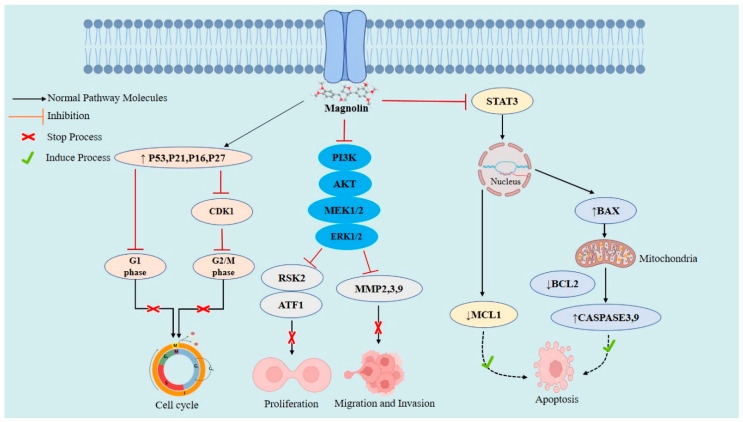
Anticancer mechanisms of magnolin.

According to various studies in the documented literature on magnolin’s anticancer action, this therapeutic substance may prevent cancer by inhibiting the cell cycle, preventing metastasis, inducing apoptosis, and suppressing cell proliferation and migration [94] (Figure 3). The effective dose and the mechanism of action may differ depending on the kind of cancer (Table 2). Furthermore, the following provides comprehensive information on magnolin’s anticancer mechanism (Figure 3).

### 2.4. Magnolin against Various Cancers

#### 2.4.1. Breast Cancer

Breast cancer is the most often diagnosed cancer and a significant cause of mortality in women worldwide [104,105]. It is the second most common cause of cancer-related deaths among women. The pathogenesis of breast cancer is a complex, multi-step process with associated factors such as multiple cell types, and its prevention remains a significant challenge globally [106,107]. It has the ability to spread to other portions of the body, such as the bone, liver, lung, and brain, making it an incurable disease in most cases. However, if the disease is detected early, the prognosis and chances of survival are generally favorable [106,108]. The treatment of breast cancer involves a multidisciplinary approach that encompasses various methods such as locoregional therapy, which involves surgery and radiation therapy, as well as systemic therapy. Systemic therapy consists of several types of treatments, including hormone therapy for chemotherapy, hormone-positive disease, anti-HER2 therapy for HER2-positive disease, and more recently, immunotherapy [109].

According to recent studies, the treatment of MDA-MB-231 breast cancer cells with magnolin suppresses proliferation and invasion and promotes apoptosis. In addition, magnolin significantly downregulated the expression of CDK1, BCL2, MMP2, and MMP9 and upregulated the cleaved caspases 3 and 9 [95]; caspase-9, which plays a crucial role in regulating apoptosis, is involved in various stimuli such as oncogenes, DNA-damaging agents, cellular stress, and apoptotic processes. Moreover, many chemotherapeutic drugs utilize caspase-9 to initiate apoptosis [95,110]. Breast cancer migration can be inhibited by preventing the phosphorylation of ERK in the affected cells [111]. The compound also diminished the phosphorylation of MEK1/2 and extracellular-signal-regulated kinase (ERK)1/2. Magnolin is known to modulate the activity of caspase proteins through the MEK-ERK-BCL2 axis. The ERK protein is situated downstream of MEK and has been identified as a significant factor in influencing tumor growth in various cancer studies [112]. In a prior study, the treatment of MDA-MB-231 breast cancer cell lines with magnolin reduced the bone loss and bone-resorbing activity caused by breast cancer through suppressed mRNA expression and the secretion of MMP-9 and cathepsin K activities. Magnolin also prevented the start and development of the “vicious cycle” between cancer metastases and the bone microenvironment by preventing PTHrP synthesis in osteoclastic bone resorption breast cancer cells [96]. When used against the cancer cell line MCF-7, Vo et al. observed that magnolin showed only moderate cytotoxic effects [57].

#### 2.4.2. Lung Cancer

GLOBOCAN, the International Agency for Research on Cancer, provided cancer statistics in 2020 worldwide, with an appraised 19.3 million new cancer cases, in which lung cancer remained the primary cancer killer [113,114]. The mechanisms and processes that underlie the development of lung cancer are intricate and not entirely comprehended [115]. The primary reason for lung cancer is tobacco smoking, and about 80% to 90% of cases are attributed to smoking cigarettes [116]. Continued exposure can cause genetic mutations, which interfere with protein synthesis and disrupt the cell cycle, ultimately promoting the development of cancer. BCL2, MYC, and p53 are the most common genetic mutations linked with small-cell lung cancer, while non-small-cell lung cancer is most commonly caused by mutations in EGFR, KRAS, and p16 [115,117,118].

The ERKs/RSK2 signaling pathway is a promising target for cancer research and therapy, as it plays a critical role in cancer cell survival, proliferation, and invasion. The inhibition of this pathway promotes cancer cell death and enhances the effectiveness of chemotherapy [119]. Additionally, targeting this pathway may have potential in cancer prevention, as it is involved in cancer initiation and progression. Overall, exploring the therapeutic potential of the ERKs/RSK2 pathway may lead to the development of effective cancer treatments and preventive measures [97,119]. Recent research has shown that magnolin can increase lung cancer cells’ chemosensitivity by preventing cell proliferation, migration, and invasion. Magnolin inhibits cell migration by inhibiting ERKs/RSK2 signaling, which NF-κB-mediated Cox-2 gene expression enhances. Moreover, findings have shown that magnolin suppressed the activity of MMP-2 and MMP-9 [97], two crucial enzymes involved in focal adhesion and cell migration [120]. Lee and colleagues showed in their previous study that magnolin prevented the phosphorylation of RSK2 and its downstream targets, including c-Jun, ATF1, and AP-1, which is a dimer composed of Jun and Fos proteins. Additionally, magnolin’s ability to block ERKs hindered EGF-induced anchorage-independent cell transformation and colony expansion of A549 human lung cancer cells and NIH3T3 cells expressing Ras (G12V) when grown in soft agar [98]. So, targeting the ERKs/RSK2 signaling pathway is an important approach for developing chemoprevention and cancer therapy.

#### 2.4.3. Liver Cancer

Regarding cancer-related mortality among men, liver cancer is the third most common [121]. It is a malignant tumor that often develops in individuals who have a history of long-term liver disease and cirrhosis, and it is characterized by its aggressive nature [122]. In cancer, numerous signaling pathways undergo changes, and in liver cancer specifically, certain pathways are observed to be disrupted. The primary pathways that play a role in the development of hepatocellular carcinoma (HCC) are those that regulate the signaling of growth factors such as insulin-like growth factor (IGF), epidermal growth factor (EGF), platelet-derived growth factor (PDGF), fibroblast growth factor (FGF), and hepatocyte growth factor (HGF/MET) [123]. The Ras/Raf/MEK/ERK and PI3K/AKT/mTOR cascades are the primary signaling pathways that are activated after the activation of receptor tyrosine kinases [124]. Tumor development, cell cycle progression, apoptosis, and survival depend on the PI3K/AKT/mTOR signaling system [54,125,126]. According to several studies, over 50% of hepatocellular carcinoma patients have an active phosphatidylinositol-3 kinase PI3K/AKT/mTOR pathway [127,128]. The PI3K-AKT/mTOR and ERK/MEK pathways were revealed to be the targets of magolin’s synergistic action with the BRAF inhibitor (SB590885) in halting the progression of liver cancer (Bel-7402 and SK-Hep1) cells in vitro [99], which are the best characterized and most frequently activated intracellular pathways in liver cancer [129]. Additionally, Llovet et al. (2008) stated that impeding the MEK/ERK pathway can lead to a reduction in cancer cell proliferation [130].

#### 2.4.4. Ovarian Cancer

Most deaths from gynecologic cancers are caused by ovarian cancer, and it is one of the most prevalent female malignancies [131,132]. Age, early menarche, null parity, late menopause, environment, and family history are risk factors [133,134,135], out of which, genetic and epigenetic factors are deemed the most significant among all the factors [136], but breastfeeding and pregnancy reduce this risk [137,138]. Individuals afflicted with this deadly illness have a mere 45.6% 5-year survival rate [139]. Patients with advanced ovarian cancer have the worst prognosis regarding curative therapeutic options, with cytoreductive surgery and cisplatin-based chemotherapy as the cornerstones of care. However, many patients show intrinsic or acquired resistance to cisplatin-based treatment regimens [140,141,142]. In this situation, magnolin therapy reduced the accumulation of G1 and G2/M cell cycle phases and the activation of ERK1/2 in TOV-112D ovarian cancer cells, resulting in the suppression of the ERK1/2-MEK pathway, a vital signaling mechanism that facilitates ligand-stimulated signals by inhibiting the initiation of cell proliferation, differentiation, and cell survival [143]. Of particular note, magnolin prevented the colony formation of TOV-112D cells on soft agar. Furthermore, in xenograft tissues, magnolin increased the proteins p16Ink4a and p27Kip1, which are essential indicators of cellular senescence [45]. Like this, magnolin, which inhibits ERK1 and ERK2 with IC_50_ values of 16 and 68 nM, restrained ovarian cancer TOV-112D cell proliferation and colony expansion. Notably, cellular senescence was induced in TOV-112 when magnolin administration reduced the phosphorylation of ATF-1 at Ser63, a downstream target of RSK2 [100].

#### 2.4.5. Prostate Cancer

Prostate cancer (PC) is the second most commonly diagnosed cancer and the second leading cause of cancer-associated deaths in men after lung cancer [144,145]. It is a complex and diverse type of cancer that can be categorized based on aggressiveness, grade, and age of onset. Exogenous factors such as diet, sexual behavior, physical activity, and occupation may influence the progression of the disease, but only three established risk factors are ethnicity, age, and heredity (first-line relatives or relatives with early-onset PC [146]. Magnolin has an IC_50_ value of 0.51 μM and is a potent antiproliferative agent against PANC-1 prostate cancer cells. Additionally, it can cause the PANC-1 cell to form suppressive colonies in a concentration-dependent manner. Furthermore, the PANC-1 cell was subjected to an inhibitory effect on migration caused by MMP3. Additionally, magnolin was substantially less hazardous to normal human cells than the growth-inhibiting positive control medication doxorubicin (6.96% and 30.48%, respectively, at 5 g/mL) [76]. In a prior study, magnolin prevented the growth and survival of the tumor in PC3 and Du145 prostate cancer cells by causing cell cycle arrest through P53/P21 activation and inducing apoptosis both in vitro and in vivo. Additionally, magnolin reduced the activation of Akt protein kinase and increased the expression of cleaved caspase3 to inhibit cell growth and promote death [43].

#### 2.4.6. Pancreatic Carcinoma

Pancreatic cancer is a form of carcinoma that originates from the cells lining the pancreatic duct, specifically known as pancreatic ductal carcinoma [147]. It is currently the fourth leading cause of cancer-associated deaths. However, due to its aggressive nature, it is expected to become the second most prevalent cause of cancer-associated deaths by 2030 [148]. The cytotoxic properties of lignans isolated from the plant Zanthoxylum alatum were investigated in pancreatic carcinoma cell lines (MIA-PaCa) and lung carcinoma cell lines (A549) using the MTT assay. The results indicated that 4′-O-demethyl magnolin extracted from Zanthoxylum alatum exhibited remarkable cytotoxic activity against these cell lines [101].

#### 2.4.7. Colorectal Cancer

Colorectal cancer is among the most commonly diagnosed malignancies worldwide, and it is one of the leading causes of cancer-associated death [149,150]. Several risk factors, including age, family history, diet, lifestyle, obesity, alcohol intake, and smoking, influence the occurrence of colorectal cancer. Environmental variables may also significantly impact the development of colorectal cancer [151,152,153]. The treatment of colorectal cancer is being revolutionized by cancer immunotherapy, which employs immune checkpoint inhibitors to empower the body’s immune system to more effectively detect and eradicate cancerous cells [154]. Autophagy improves the efficacy of immunotherapy by facilitating the optimal release of immunostimulatory signals. This is achieved through the delivery of antigens to immune cells, such as antigen-presenting cells and CD8+ cytotoxic T lymphocytes [155]. Magnolin inhibits cell growth and causes cell cycle arrest in HCT116 and HT29 cells in the G2/M phase [102]. Similarly, by encouraging autophagy and cell cycle arrest via the LIF/Stat3/Mcl-1 pathway, magnolin inhibits the formation of tumors in HCT116 and SW480 colorectal cancer cells in vitro and in vivo without exhibiting any observable adverse effects [103]. Recent data from various sources also indicate that controlling autophagy may offer crucial insights into halting the growth and spread of cancer and improving cancer treatments [156,157,158]. The biological efficacy of lignans, which includes magnolin derived from the aboveground portions of Artemisia gorgonum Webb, has been examined to determine their ability to destroy cells in different types of mammalian tumor cell lines and in normal murine cells (CFU-GM). The findings showed that magnolin exhibited only slight potency and selectivity against murine colon, and there was no significant evidence of its ability to destroy human tumor cells [44].

### 2.5. Pharmacokinetic Features

Pharmacokinetics (PKs) plays a crucial role in drug discovery by helping to improve the absorption, distribution, metabolism, and excretion (ADME) properties of potential drug candidates. The ultimate aim is to create a clinical candidate that achieves the desired efficacy and safety profile by attaining a concentration–time profile in the body that is appropriate [159,160,161]. Assessing the PKs of herbal medicinal drugs, including the extent of oral bioavailability, can help to establish a connection between pharmacological data and clinical effects. It can also facilitate the development of appropriate dosage regimens by providing valuable information for rational drug design [160,162]. Pharmacokinetics and pharmacogenetics are significant factors in drug discovery that impact the success of treatment. In the case of cancer treatment, these factors are crucial due to the elevated levels of toxicity associated with many cancer medications. Therefore, comprehending how drugs are metabolized, how they interact with their targets, and how genetic variations can impact drug response is crucial for developing effective and safe treatments for cancer [163]. Various in vivo studies revealed the PK properties necessary to understand the ADME of magnolin.

In 2010, Kim and colleagues first conducted an experiment using live rats to investigate the PKs of magnolin, which is the major active phytochemical in Magnolia fargesii. They administered the compound intravenously and orally in various doses and found that the oral bioavailability ranged from 54.3% to 76.4% for the doses they tested. They also discovered that magnolin bound strongly to plasma proteins in rats, with a consistent binding value of 71.3% to 80.5% across a concentration range of 500 to 10,000 ng/mL. The researchers found that the PKs of magnolin were not affected by the dosage, regardless of whether it was administered intravenously or orally [164]. The process of drug metabolism is a critical aspect of pharmacokinetics and plays a significant role in drug development [165]. Studies have shown that in rats, magnolin undergoes metabolism to produce 4’-O-desmethyl magnolin, 4″-O-desmethyl magnolin, and their respective glucuronides [166]. Another study reported that Aspergillus niger selectively metabolizes magnolin to produce 4’-desmethyl magnolin and 4’-desmethyl-5’-hydroxymagnolin [167]. According to Kim et al. (2011), magnolin is predicted to be metabolized into its oxidative metabolites by several CYP enzymes, including CYP2C8, CYP2C19, CYP2C9, and CYP3A4. As a result, there is potential for drug interactions when co-administered with drugs that can inhibit or diminish CYP2C enzymes [168]. In cases of elimination, magnolin is rapidly absorbed when administered orally and is almost entirely eliminated through metabolic processes [164].

## 3. Methodology

### 3.1. Literature Searching Strategy

An up-to-date (until March 2023) search was made in the various databases, such as PubMed, Springer Link, Wiley Online, Web of Science, ScienceDirect, and Google Scholar, with the common keyword “magnolin”, which was then paired with “cancer,” “tumor,” “anticancer activity,” “antiproliferation activity,” “cytotoxic activity,” “antitumor activity,” “human cancer,” “biological activities,” “pharmacological effects,” “pharmacological activities,” “biological sources,” “chemical features,” “in vivo studies”, or “in vitro studies “. No time and language restrictions were imposed in the search criteria. The studies were evaluated in-depth, with information regarding the sources, dose/concentration, test system, proposed action mechanism, and overall conclusion and recommendation.

### 3.2. Inclusion Criteria

1. Studies carried out in vitro, ex vivo, or in vivo with or without utilizing laboratory animals, including mice, rats, rabbits, and humans, and their derived tissues or cells.
2. Studies with anticancer activities and botanical sources of magnolin.
3. Studies with magnolin or its derivatives or preparations.
4. Magnolin or its derivatives provide joint activity with other chemical compounds.
5. Studies which do or do not imply possible mechanisms of action.

### 3.3. Exclusion Criteria

1. Studies demonstrated data duplication and titles and/or abstracts not meeting the inclusion criteria.
2. Magnolin in other studies not covering the current issue.
3. Papers written in languages other than English.
4. Studies without full text available.
5. Case reports, letters, editorials, and commentaries

## 4. Conclusions

Cancer remains one of the most fearsome diseases in the world, causing a significant number of deaths every year. Among the other strategies, chemotherapeutic approaches are considered one of the most potential and effective therapeutic modalities in cancer. Natural products and their derivatives are the potential sources of anticancer lead compounds. This review suggests that, to date, magnolin has been isolated from 23 medicinal plants under 13 genera. Magnolin between 10 to 125 μM showed anticancer effects against breast, lung, liver, ovarian, prostate, and colon cancers, while its derivative, 4′-O-demethyl magnolin, is evidently effective against lung and pancreatic cancers. Magnolin exerts anticancer effects against various cancer cell lines or systems through diverse mechanisms that include cytotoxicity, apoptotic cell death, cell cycle arrest, antimigratory, antiproliferative, and anti-invasion pathways. However, according to the database reports, more work has been done on its anticancer effects against breast and lung cancers. The cytotoxicity, apoptosis, and antiproliferative effects of magnolin were predominant among the anticancer mechanisms. More research is necessary on this hopeful anticancer lead compound.

## Figures and Tables

**Table 1:** Biological sources of magnolin.

Plant Name	Plant Part	References
*Magnolia fargesii*	Flower buds	[37,52]
*Zanthoxylum simulans*	Bark	[53]
*M. biondii*	Flower buds	[54,55,56]
*Pseuderanthemum carruthersii*	Root	[57]
*M. officinalis*	Flower buds	[58]
*M. sargentiana* and *M. sprengeri*	-	[42]
*Castilleja tenuiflora*	Aerial part of roots	[59]
*Astrantia major*	Fruits	[60]
*Artemisia gorgonum* Webb	Leaves and flowers	[44]
*Acorus calamus*	-	[61]
*Rollinia mucosa*	Seeds	[62]
*M. denudata*	Flowers	[63,64]
*M. kobus*	Flower buds, bark	[65,66]
*M. salicifolia*	Flower buds	[65]
*Licaria armeniaca*	Trunk wood, fruits	[67,68]
*Achillea holosericea.*	Aerial parts	[69]
*Annona pickelii*	Leaves	[70]
*M. liliflora*	Leaves	[71]
*A. gypsicola*	Aerial parts and roots	[72,73]
*Z. alatum*	Seeds	[74]
*Hernandia ovigera*	Leaves	[75]
*Physalis peruviana*	Fruits	[76]

**Table 2:** Pharmacological mechanisms of magnolin involved in its anticancer activities.

Type of Cancer	Experimental Model/ Cell Line	Tested Concentrations	Efficacy, IC_50_ (Exposure Time)	Anticancer Effects and Mechanisms	Reference
Breast cancer	MDA-MB-231	20 and 50 µM	30.34 µM (24 h)	↓MEK1/2, ↓ERK1/2, ↓CDK1, ↓BCL2, ↓MMP 2 and 9, ↑CASPASES3 and 9 ↓proliferation, ↓invasion, ↑apoptosis	[95]
	MDA-MB-231	10, 20, 40, 60, 80, and 100 µM	-	↓mRNA expression, ↓PTHrP, ↓MMP9, ↓cathepsin K, ↑RANKL/OPG ratio ↓bone loss and bone-resorbing activity	[96]
-	MCF-7	100 µg/mL	-	↑cytotoxicity	[57]
Lung cancer	JB6CL41, NCI-H1975 and A549	15, 30, and 60 µM	-	↓ERK1/2, ↓MMP2, ↓MMP9, ↓RSK2, ↓migration, ↓invasion	[97]
	NIH3T3, A549	15, 30, and 60 µM	-	↓ERK1, ↓ERK2, ↓RSK2, ↓ATF1, ↓AP1, ↓proliferation, ↓migration	[98]
Hepatocellular carcinoma	BEL-7402 and SK-HEP1	25, 50, 75 100, and 125 μM	-	↓MEK, ↓PI3K, ↓AKT, ↓proliferation	[99]
Ovarian cancer	TOV-112D	15, 30, and 60 µM	-	↓ERK1, ↓ERK2, ↑P^16Ink4a^, ↑P^27Kip1^, ↓G1 and G2/M phase, ↓proliferation	[45]
	TOV-112D	-	16 nM 68 nM	↓ERK1, ↓ERK2, ↓ATF1, ↓RSK2 ↓proliferation	[100]
Prostate cancer	PANC-1	-	0.51 µM	↓MMP3, ↓proliferation, ↓migration	[76]
	PC3 and DU145	50 and 100 µM	-	↓AKT, ↓BCL2, ↑BAX, ↑CASPASE3, ↑P53, ↑P21 ↓G1, G2 phase, ↑apoptosis	[43]
Pancreatic carcinoma	MIA-PaCa	-	-	↑cytotoxicity	[101]
Colon cancer	HCT116 and HT29	-	-	↓G_2_/M-phases, ↓proliferation	[102]
Colorectal cancer	HCT116 and SW480	10, 20, 30, and 40 µM	-	↓LIF, ↓STAT3, ↓MCL1 ↑ autophagy ↓cell cycle	[103]

Arrows (↑ and ↓) show an increase or decrease in the obtained variables. MEK1/2: mitogen-activated protein kinase kinase 1 and 2; ERK1/2: extracellular signal-regulated kinase 1 and 2; CDK1: cyclin-dependent kinase 1; BCL2: B-cell lymphoma 2; MMP 2 and 9: matrix metalloproteinases 2 and 9; RSK2: ribosomal S6 kinase 2; ATF1: activating transcription factor 1; AP1: activator protein 1; PI3K: phosphoinositide 3-kinase; AKT: protein kinase B; BAX: Bcl-2-associated X protein; LIF: leukemia inhibitory factor; STAT3: signal transducer and activator of transcription 3; MCL1: myeloid leukemia 1; P53: tumor protein P53; P21: tumor protein P21.

## Data Availability

The processed data are available from the corresponding author upon request.
